# Diagnosis of sleep-disordered breathing using few-shot learning

**DOI:** 10.3389/fpubh.2026.1892597

**Published:** 2026-08-14

**Authors:** Cheng Jiao, Ying Tao, Yiyang Zhao, Jing Pei, Jing Li, Bing Guan, Yawen Shi

**Affiliations:** 1 Department of Otorhinolaryngology Head and Neck Surgery, Northern Jiangsu People's Hospital Affiliated to Yangzhou University, Yangzhou, Jiangsu, China; 2 Department of Otorhinolaryngology Head and Neck Surgery, Northern Jiangsu People's Hospital, Yangzhou, Jiangsu, China; 3 Department of Blood Purification Center, Northern Jiangsu People's Hospital, Yangzhou, Jiangsu, China; 4 Department of Information and Artificial Intelligence, Yangzhou University, Yangzhou, Jiangsu, China; 5 Department of Otolaryngology, Head and Neck Surgery, The Affiliated Jiangning Hospital with Nanjing Medical University, Nanjing, Jiangsu, China; 6 Department of Otorhinolaryngology, The First Affiliated Hospital with Nanjing Medical University, Nanjing, Jiangsu, China

**Keywords:** contrastive learning, few-shot learning, medical text classification, prompt learning, sleep-disordered breathing

## Abstract

Sleep-Disordered Breathing (SDB) is a common and clinically significant disorder characterized by recurrent airflow limitation and oxygen desaturation during sleep, which can lead to serious cardiovascular and metabolic complications. Accurate and early diagnosis of SDB is crucial for timely clinical intervention and risk stratification, yet diagnostic results are often influenced by substantial variations in physicians' clinical experience and diagnostic skills across different regions, particularly in large-scale screening and real-world medical settings. However, existing diagnostic methods based on traditional machine learning or fine-tuned deep models often suffer from limited labeled data, poor generalization in few-shot scenarios, and insufficient exploitation of medical domain knowledge. To address these challenges, in this paper, we propose a few-shot method that integrates prompt learning with contrastive learning for SDB diagnosis, short for SDB-FL. Specifically, SDB-FL employs a manual prompting strategy based on handcrafted templates, together with a knowledgeable verbalizer that incorporates medical domain knowledge, to activate latent domain knowledge embedded in pre-trained language models, thereby enabling effective task adaptation under data-scarce conditions. Meanwhile, contrastive learning is introduced to enhance the discriminative ability of representations by promoting intra-class compactness and inter-class separability at the semantic level. Experimental results on both English and Chinese datasets demonstrate that our SDB-FL consistently outperforms strong baseline methods across multiple few-shot settings.

## 1 Introduction

Sleep-Disordered Breathing (SDB) is a prevalent sleep-related respiratory disorder encompassing conditions such as obstructive sleep apnea (OSA), characterized by recurrent episodes of partial or complete upper airway obstruction during sleep, leading to intermittent hypoxemia and sleep fragmentation (1). Extensive clinical studies have shown that untreated SDB is closely associated with a wide range of adverse health outcomes, including cardiovascular disease, metabolic dysfunction, cognitive impairment, and increased mortality risk (2, 3). For example, Recent epidemiological analyses estimate that obstructive forms of SDB affect tens of millions of adults in high-income countries and may impact over 30 % of middle-aged adults in general populations (4).

Accurate and timely diagnosis of SDB is critical to clinical intervention and risk stratification (5). In real-world practice, both the gold-standard polysomnography (PSG) and clinical judgment are constrained by variations in physician expertise and unequal access to diagnostic resources across different regions and care settings (6). Emerging works in the sleep medicine field highlight that a significant proportion of individuals with clinically meaningful SDB remain undiagnosed or misdiagnosed due to these constraints, leading to delayed treatment and missed opportunities for early risk stratification and intervention (7). Moreover, advances in home sleep testing and AI-assisted monitoring tools illustrate potential pathways to expand access to SDB screening and facilitate clinical decision support, yet also emphasize the need for reliable screening and decision-support methods to ensure accurate clinical decision-making across diverse healthcare contexts (8).

Despite advances in diagnostic approaches, existing methods for SDB diagnosis face significant challenges that limit their clinical effectiveness and scalability. On the one hand, the current gold standard, in-laboratory PSG, is resource-intensive, costly, and uncomfortable, often requiring specialized facilities and expert interpretation, which restricts its accessibility for large-scale or routine screening. On the other hand, existing AI-based methods have shown promise results on SDB detection from physiological signals and wearable sensors, but their performance often vary widely across different population groups, indicating limited generalizability and robustness in real-world clinical settings. More importantly, existing widely used machine learning models depend on engineered features or require extensive labeled PSG data, which is costly and time-consuming to obtain, making it difficult to deploy effective diagnostic systems in low-resource environments. Consequently, there remains an urgent need for reliable screening and decision-support methods that can generalize effectively under limited data conditions and provide interpretable assistance for clinicians across diverse healthcare contexts.

In this paper, we propose a novel few-shot learning method that integrates prompt learning with contrastive learning for robust SDB diagnosis (SDB-FL). Few-shot learning has gained increasing attention in medical NLP and clinical classification tasks because it enables effective model adaptation with only a small number of annotated examples, addressing the data scarcity problem inherent in clinical settings (9). Existing prompt-learning methods mainly improve downstream task adaptation by optimizing prompt representations, whereas contrastive-learning approaches enhance feature discrimination through representation learning. Although both techniques have demonstrated promising performance in few-shot learning, they are generally investigated independently or applied to general NLP tasks and specific medical applications. However, their joint application to few-shot medical short-text classification for sleep-disordered breathing (SDB) diagnosis remains largely unexplored, where labeled data are scarce and discriminative semantic representations are essential. Motivated by this gap, we develop SDB-FL to jointly leverage the complementary strengths of prompt learning and supervised contrastive learning within a unified framework. In our SDB-FL, prompt learning is introduce to bridge the gap between pre-training objectives and downstream classification tasks by reformulating classification into a masked language prediction problem, which can leverage rich domain knowledge embedded in pre-trained model to reduce the reliance on extensive labeled corpora. Meanwhile, contrastive learning has been demonstrated to significantly enhance representation quality by pulling semantically similar samples closer and pushing dissimilar ones apart, which can mitigate overfitting and improve class separability in few-shot scenarios (10). By combining a discrete prompting strategy consisting of multiple manual templates with a knowledgeable verbalizer and a supervised contrastive objective, our SDB-FL effectively activates latent medical knowledge while learning discriminative representations, which enable consistent performance even with limited task-specific data. Extensive experiments on both English and Chinese datasets demonstrate that our SDB-FL consistently outperforms strong baseline methods across multiple few-shot settings. The main contributions are summarized as follows:

- We propose SDB-FL, a novel few-shot learning framework for sleep-disordered breathing diagnosis, which integrates prompt learning with supervised contrastive learning to address data scarcity and limited generalization in clinical settings.
- For prompt learning, we design a manual prompting strategy with handcrafted templates and a knowledgeable verbalizer to effectively activate latent domain knowledge within the pre-trained model.
- We introduce supervised contrastive learning to enhance the discriminability of representations by improving intra-class compactness and inter-class separability.
- Extensive experiments on both Chinese and English medical datasets demonstrate that our SDB-FL consistently outperforms strong baselines across multiple few-shot settings.

## 2 Related work

### 2.1 Sleep-disordered breathing

Polysomnography (PSG) is widely recognized as the gold standard for the diagnosis of Sleep-Disordered Breathing, which provides comprehensive physiological monitoring of airflow, respiratory effort, oxygen saturation, electroencephalography, electrocardiography, and other sleep-related parameters throughout the night (11). Standardized scoring criteria established by the American Academy of Sleep Medicine (AASM) ensure objective assessment of apnea and hypopnea events and enable calculation of key indices such as the apnea-hypopnea index (AHI), which remains central to clinical diagnosis and severity stratification (12). Despite its diagnostic reliability and standardized workflow, PSG is expensive, labor-intensive, and dependent on specialized sleep laboratories and trained technicians, which limits accessibility and scalability (13). The requirement for manual scoring and expert interpretation further introduces potential inter-rater variability and regional disparities in diagnostic practice (6). These operational and resource constraints restrict the feasibility of PSG for large-scale screening and early detection.

To address these limitations, increasing attention has been directed toward machine learning and AI-based approaches for automated SDB detection. Recent systematic reviews report that AI models leveraging physiological signals, such as electrocardiogram (ECG), electroencephalogram (EEG), photoplethysmography (PPG), and oximetry, can achieve promising diagnostic performance in apnea detection and severity estimation (5). Wearable sensor-based systems combined with AI algorithms have further expanded the possibility of home-based and continuous sleep monitoring, which can offer more accessible alternatives to laboratory PSG (7). Recently, due to the powerful feature learning capability, deep learning architectures, including convolutional and recurrent neural networks, have demonstrated effectiveness in sleep stage classification and event detection from multivariate time-series data. For example, Phan et al. proposed an attention-based recurrent neural network for automatic sleep stage classification using single-channel EEG signals, which validated that deep sequential modeling can effectively capture temporal dependencies in sleep dynamics and improve classification performance without relying on extensive handcrafted features (14). Similarly, Chambon et al. developed a deep learning architecture for temporal sleep stage classification using multivariate and multimodal time-series data, the convolutional neural network is introduced to learn hierarchical representations from raw physiological signals and achieve competitive performance compared to traditional feature-engineered approaches (15). Although these deep learning methods illustrate the potential of automated sleep analysis, their performance often depends on large-scale annotated datasets and carefully controlled experimental settings. In practice, model robustness may degrade when applied to heterogeneous populations or limited data scenarios, underscoring ongoing challenges in generalization, data efficiency, and clinical deployment.

### 2.2 Few-shot learning

Few-shot learning has emerged as a critical paradigm for addressing scenarios where labeled data are scarce but rapid generalization to new tasks is necessary, such as in clinical text classification and medical diagnosis (16). Instead of relying on large annotated corpora, few-shot learning approaches enable models to leverage prior knowledge and adapt to novel classes with only a handful of examples, reducing the annotation burden and improving applicability in real-world, low-resource settings. This necessity is especially pronounced in medical domains, where obtaining expert-labeled data is costly and time consuming. Recent surveys emphasize that few-shot learning techniques span a variety of strategies, including meta-learning, transfer learning, and leveraging pre-trained representations to enable fast adaptation to new tasks with minimal labeled samples (17). Moreover, the rise of pre-trained language models (PLMs) has further fueled interest in few-shot learning by demonstrating that models can exploit rich pre-trained knowledge to perform downstream tasks with limited supervision (18).

Among the main methodological advances in few-shot learning, prompt learning reformulated downstream tasks into formats closely aligned with the objectives in PLM, which allowed models to “fill in the blank” guided by task-specific prompts, significantly improving performance with few examples (20). For example, Zhu et al. proposed a soft knowledgeable prompt learning method for short text classification, which considered both the template generation and classification performance to construct prompts for label prediction (19). Zhu et al. proposed a prompt-learning method for short text classification, which improved classification performance under sparse and highly ambiguous text conditions (20). Li et al. enhanced model robustness by integrating domain adaptation and knowledge-optimized verbalizers into prompt learning (21). Wang et al. proposed a multi-modal soft prompt learning method, which jointly modeled the textual and image information into a continuous prompt embedding as the input of PLMs (22). Xiao et al. proposed a multi-modal prompt-tuning method for thyroid nodule ultrasound diagnosis, which jointly utilized ultrasound images and textual descriptions to construct prompt-guided multi-modal representations and effectively improve diagnostic performance (23). Besides prompt learning, contrastive learning methods aim to enhance representation discriminability by pulling semantically similar examples closer and pushing dissimilar ones apart, which has been shown to improve generalization from limited data (24). The representative works such as ConsPrompt explore multi-degree contrastive samples to strengthen prompt robustness across classes (25). Additional extensions include adversarial knowledge-stimulated contrastive prompting, which enriches prompt-based adaptation by integrating contrastive learning with task-specific knowledge stimulation to improve performance on few-shot natural language understanding tasks (26). Overall, these advances demonstrate that combining prompt tuning with contrastive objectives can substantially mitigate the challenges of limited labeled data and improve model adaptability in few-shot scenarios.

## 3 Methodology

### 3.1 Motivation

In recent years, artificial intelligence based medical text classification methods have been widely applied in various clinical diagnosis and healthcare analysis tasks. However, in real world clinical scenarios, obtaining large scale high quality annotated medical texts is extremely expensive and time consuming, since it usually requires professional medical knowledge and expert annotation. Consequently, most practical medical natural language processing tasks suffer from limited labeled data, especially in specialized clinical scenarios.

Although existing pretrained language model based medical text classification methods have achieved good performance, several challenges still remain under few shot settings. On the one hand, traditional fine tuning methods strongly rely on sufficient annotated samples and are likely to suffer from overfitting when the training data are limited. On the other hand, existing prompt learning methods mainly reformulate downstream tasks as masked language modeling tasks for classification, but they often fail to explicitly improve the discriminability of semantic representations across different categories. As a result, clinically similar samples are still difficult to distinguish, which leads to unstable decision boundaries and limited generalization capability.

To address the above challenges, this paper proposes a few shot framework for sleep-disordered breathing classification by combining prompt learning with supervised contrastive learning. Specifically, a prompt based learning strategy together with knowledgeable verbalizers is first introduced to better utilize the semantic knowledge embedded in pretrained language models, thereby improving the model adaptability under limited data conditions. Subsequently, a supervised contrastive learning module is incorporated to enhance the discriminability of semantic representations by pulling samples from the same category closer and pushing samples from different categories farther apart. Finally, prompt based representation learning and supervised contrastive learning are jointly integrated to further improve the robustness and generalization capability of the model for few shot medical text classification tasks. Experimental results on both Chinese and English medical datasets demonstrate that the proposed method achieves stable and effective classification performance under multiple few shot settings and maintains good generalization ability across different medical text distributions.

### 3.2 Overall architecture

**Figure 1:**
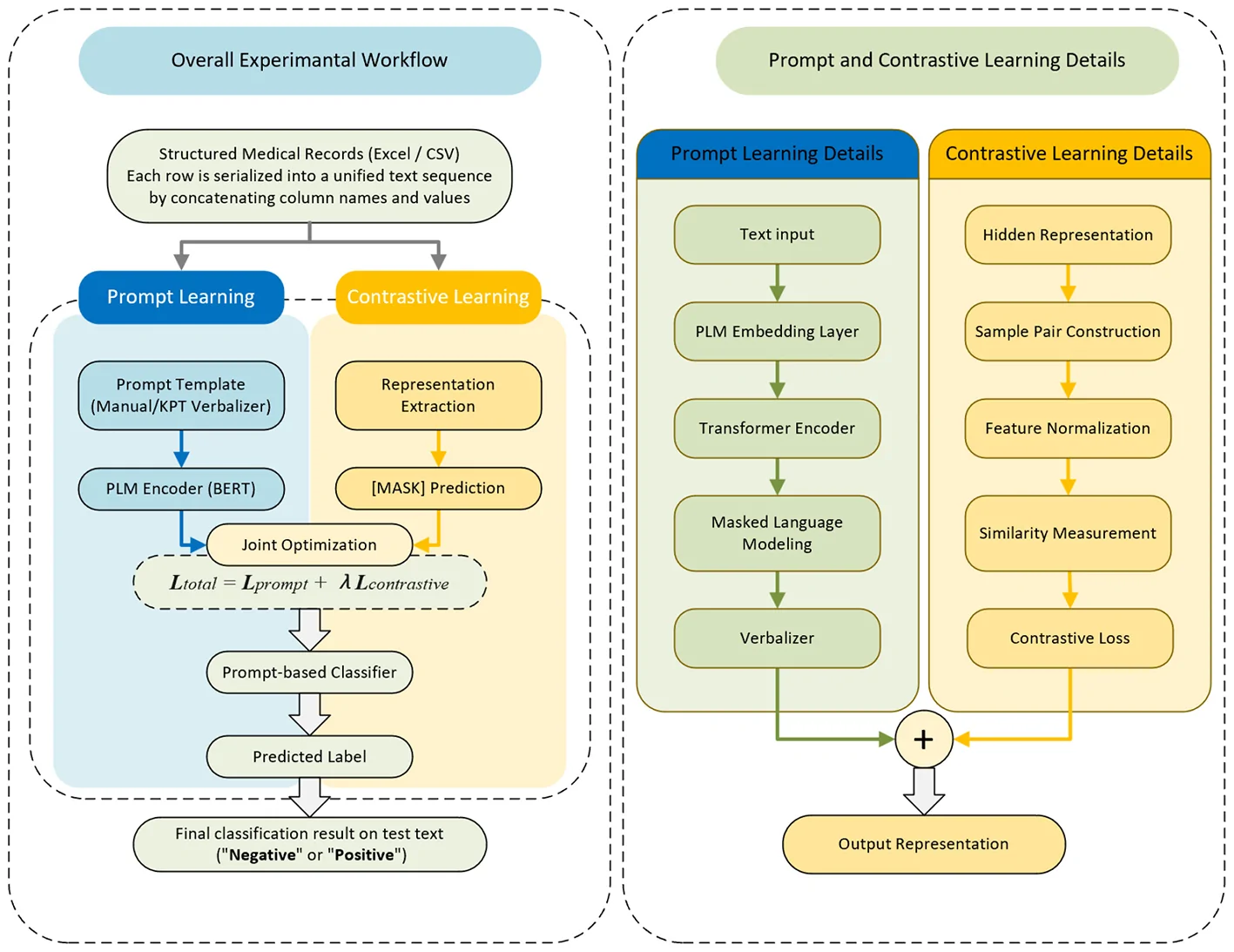
Illustration of the overall architecture of the proposed method. The left part shows the main framework, while the right part details the structures of prompt learning and contrastive learning, corresponding to the dashed box in the middle on the left.

Medical text based diagnosis of sleep disordered breathing can be formulated as a binary classification task, whose main objective is to determine whether a patient suffers from sleep disordered breathing according to clinical descriptions and medical indicators. To improve semantic representation capability and classification robustness under limited labeled data conditions, this paper proposes a few shot learning framework named SDB FL. As illustrated in Figure 1, the proposed framework first reformulates the downstream task as a prompt based masked language modeling task. Subsequently, a designed prompt template together with a knowledgeable verbalizer is introduced to better utilize the latent domain knowledge embedded in pretrained language models, thereby improving task adaptation capability under data scarce conditions. The constructed prompted text sequence is then fed into a pretrained language model for semantic representation learning. To further improve the discriminability of semantic representations, supervised contrastive learning is incorporated to model semantic relationships among samples by enhancing intra class compactness and inter class separability. Finally, the learned semantic representations are fed into a classification head to obtain the final prediction results. Specifically, the proposed framework mainly consists of the following components:

**Prompt-based Representation Learning:** To effectively leverage semantic priors embedded in pretrained language models, the original medical text is first reformulated into a prompt-based input sequence. Specifically, a prompt template is introduced into the clinical text to construct a prompt-guided masked language modeling input. Meanwhile, a knowledgeable verbalizer is designed to establish semantic associations between category labels and domain-related medical label words. The prompted sequence is then fed into a pretrained language model to obtain contextual semantic representations, and the semantic feature corresponding to the prompt position is extracted as the representation of the input sample for downstream classification. This strategy enables the pretrained language model to better adapt to few-shot medical text classification tasks under limited labeled data conditions.

**Supervised Contrastive Learning:** To further enhance the discriminability of semantic representations, supervised contrastive learning is incorporated during training. Specifically, samples belonging to the same category within a mini-batch are regarded as positive pairs, while samples from different categories are treated as negative pairs. By encouraging semantically similar samples to be closer and dissimilar samples to be farther apart in the embedding space, the proposed framework improves intra-class compactness and inter-class separability, thereby enhancing representation robustness and generalization capability under few-shot settings.

**Classification:** Finally, the semantic representations learned by the pretrained language model are fed into a classification head to predict the probability distribution of sleep-disordered breathing categories. Through the integration of prompt-based representation learning and supervised contrastive learning, the proposed SDB-FL framework achieves robust and discriminative performance for few-shot medical text classification tasks.

### 3.3 Prompt-based representation learning

Given the *b*-th medical text sample, the original input sequence can be represented as Equations 1–5


$$\begin{array}{l} X_{b}=\left\{x_{b,1},x_{b,2},\ldots ,x_{b,N}\right\} \end{array}$$ (1)

where *N* denotes the number of tokens in the input text and *x*_*b, i*_ represents the *i*-th token.

To reformulate the downstream classification task into a prompt-based learning paradigm, a prompt template is introduced to reconstruct the original medical text:


$$\begin{array}{l} X_{b}^{p}=T\left(X_{b}\right) \end{array}$$ (2)

where *T*(·) denotes the prompt template function that inserts task-related textual patterns and the [MASK] token into the original sequence, and $X_{b}^{p}$ represents the prompted medical text.

Meanwhile, a knowledgeable verbalizer is constructed to establish semantic associations between category labels and domain-related medical label words:


$$\begin{array}{l} V=\left\{v_{1},v_{2},\ldots ,v_{C}\right\} \end{array}$$ (3)

where *V* denotes the verbalizer set and *C* is the number of categories.

The prompted sequence is then fed into a pretrained language model (PLM) to obtain contextual semantic representations:


$$H_{b}=PLM\left(X_{b}^{p}\right)$$ (4)

where $H_{b}\in {\mathbb{R}}^{N\times d}$ denotes the contextual hidden representations generated by the pretrained language model, and *d* represents the hidden feature dimension. Specifically, the hidden representation corresponding to the [MASK] token is extracted as the semantic representation of the input sample:


$$\begin{array}{l} h_{b}=H_{b}^{\left[\text{MASK}\right]} \end{array}$$ (5)

Through the prompt reformulation process, the pretrained language model can better leverage semantic priors embedded in medical corpora under few-shot settings.

### 3.4 Supervised contrastive learning

To further enhance the discriminability of semantic representations, supervised contrastive learning is incorporated during training. First, the semantic representation is projected into the contrastive feature space through a nonlinear projection head (Equations 6–8):


$$\begin{array}{l} z_{b}=g\left(h_{b}\right) \end{array}$$ (6)

where *g*(·) denotes the projection head and *z*_*b*_ represents the projected feature representation. The cosine similarity between two feature representations is defined as


$$sim\left(z_{i},z_{j}\right)=\frac{z_{i}^{⊤}z_{j}}{z_{i}z_{j}}$$ (7)

For samples belonging to the same category, the proposed framework aims to pull their feature representations closer in the embedding space, while samples from different categories are pushed farther apart. The supervised contrastive loss is formulated as


$$L_{sup\;}=\overset{i=1}{\underset{B}{\sum \text{​}}}\frac{-1}{\left|P(i)\right|}\underset{p\in P(i)}{\sum \text{​}}\log\frac{\exp\left(sim\left(z_{i},z_{p}\right)/\tau \right)}{\underset{a\in A(i)}{\sum }\exp\left(sim\left(z_{i},z_{a}\right)/\tau \right)}$$ (8)

where *P*(*i*) denotes the set of positive samples sharing the same label as sample *i*, *A*(*i*) represents all samples in the mini-batch except itself, τ is the temperature coefficient, and *B* denotes the mini-batch size.

By optimizing the supervised contrastive objective, the proposed framework improves intra-class compactness and inter-class separability, thereby enhancing representation discriminability and generalization capability under few-shot settings.

### 3.5 Classification

After semantic representation learning and supervised contrastive learning, the learned semantic representation is fed into a multilayer perceptron (MLP) classifier for downstream prediction Equations 9 and 10:


$$O_{b}=MLP\left(h_{b}\right)$$ (9)

where *o*_*b*_ denotes the output logits of the classifier.

The final category probability distribution is obtained through the Softmax function:


$${\hat{y}}_{b}=Softmax\left(O_{b}\right)$$ (10)

where ŷ_*b*_ denotes the predicted probability distribution of sleep-disordered breathing categories.

Through the collaborative optimization of prompt-based representation learning and supervised contrastive learning, the proposed framework can achieve more robust and discriminative semantic representations for few-shot medical text classification.

## 4 Experiments

### 4.1 Datasets

Two medical text classification datasets are utilized in this study, namely the English medical dataset *Medical_en* and the Chinese medical dataset *Medical_zh*. Both datasets are formulated as binary classification tasks for Sleep-Disordered Breathing, and their statistical information is summarized in Table 1. To improve the transparency and reproducibility of the experiments, we further describe the datasets in terms of data sources, sample distribution, annotation procedures, and ethical considerations. It should be noted that during data preprocessing, diagnosis-related physiological indicators, including AHI and other sleep monitoring parameters, were excluded from the model input and were not used for model training or inference to avoid potential information leakage.

**Table 1:** Statistics of the datasets.

Dataset	Train	Test	Total	#Classes
Medical_en	351	149	500	2
Medical_zh	326	119	445	2

**Medical_en**^1^**:** This dataset was collected from a publicly available sleep-disordered breathing detection dataset released on the Kaggle platform and contains 500 records, including 369 SDB-positive cases and 131 SDB-negative cases. It includes both structured clinical variables and unstructured electronic health record (EHR) text, covering demographic information, medical history, sleep habits, comorbidities, symptom descriptions, and objective clinical indicators such as blood oxygen saturation (SpO_2_), snoring patterns, and heart rate. Since this is a publicly available dataset, all personally identifiable information was removed before its release, and no additional ethical approval was required for its secondary use.

**Medical_zh:** This dataset was collected from real clinical records at Northern Jiangsu People's Hospital and manually organized by the research team. It contains 445 records, including 392 SDB-positive cases and 53 SDB-negative cases. The dataset includes demographic information, clinical symptom descriptions, medical history, lifestyle information, otolaryngology (ENT) examination records, and objective clinical indicators including the apnea–hypopnea index (AHI), minimum SpO_2_, mean SpO_2_, oxygen desaturation index (ODI), and mean heart rate. The diagnostic labels were assigned according to the corresponding clinical diagnosis recorded in the medical records. Prior to analysis, all patient-identifiable information was anonymized, and the study complied with the relevant institutional requirements for data privacy protection and research ethics.

To improve transparency and reproducibility, we explicitly describe all input variables used in this study. Across both the Medical_en and Medical_zh datasets, the input features include demographic information, physiological measurements, sleep-related indicators, oxygen saturation (SpO_2_), oxygen desaturation index (ODI), and other clinical examination results. These structured variables are converted into textual format by concatenating feature names with their corresponding values, forming natural language-like clinical descriptions as model input. This unified serialization strategy is applied consistently across both datasets.

Importantly, these variables are recorded clinical measurements and are not used as deterministic rules for label construction. The ground-truth SDB labels are determined based on standard clinical diagnostic criteria and expert annotation, rather than being directly derived from any single input variable. Therefore, no explicit label-defining or leakage-inducing features are included in the model input.

### 4.2 Baselines

To validate the effectiveness of the proposed method, several representative medical text models were selected as baseline methods for systematic performance comparison:

**TextCNN** (27): This method is based on a convolutional neural network architecture, which captures local *n*-gram features in text using convolutional kernels of different sizes, followed by max-pooling operations to extract salient information. Due to its simple structure and stable feature extraction capability, TextCNN has been widely adopted as a classical baseline for medical text classification tasks.

**TF-IDF** (28): TF-IDF is a widely used statistical text representation method that measures the importance of words based on term frequency and inverse document frequency. It provides sparse lexical features without contextual semantic information and is commonly employed as a traditional baseline for medical text classification tasks. In our implementation, TF-IDF features were extracted using Scikit-learn's TfidfVectorizer and classified using a Logistic Regression classifier with the liblinear solver, class_weight=balanced, and max_iter=200. All other parameters were kept as their default settings.

**ChineseMedicalBERT** (29): This model is a domain-specific pretrained language model for Chinese medical texts. It is pretrained on large-scale Chinese clinical corpora, enabling effective modeling of medical terminology and domain-specific semantics, thereby improving performance on downstream medical text classification tasks.

**LLaMA** (30): LLaMA is a family of large language models proposed by Meta, which is trained on large-scale publicly available text corpora to achieve strong language understanding and generation. By adopting an efficient transformer-based architecture and emphasizing high-quality data scaling, it attains competitive performance compared with larger proprietary models while maintaining relatively lower computational cost, demonstrating strong generalization and adaptability across diverse NLP tasks such as reasoning, summarization, and dialogue. In our experiments, the pretrained LLaMA-7B model was directly used for prompt-based text classification without parameter fine-tuning. During inference, text generation was performed with a temperature of 0.1. The generated responses were normalized and matched to the predefined class labels to obtain the final predictions. The model was evaluated using the same data splits and evaluation protocol as the proposed method to ensure a fair comparison.

**P-Tuning** (31): P-Tuning is a parameter-efficient prompt learning method proposed for adapting pre-trained language models to downstream tasks. By introducing trainable continuous prompt vectors and optimizing them while keeping the backbone model frozen, it enables effective task adaptation with significantly reduced training cost, achieving competitive performance on various NLP tasks such as classification, generation, and knowledge probing.

**ClinicalBERT** (32): ClinicalBERT is a domain-adapted variant of BERT that is pretrained on clinical notes from the MIMIC dataset. This model effectively captures domain-specific expressions and contextual semantics in clinical texts, leading to improved performance in clinical text classification tasks.

**MP-UDTN** (23): MP-UDTN is a multimodal prompt-tuning framework originally designed for ultrasound diagnosis of thyroid nodules. In the original model, ultrasound images and textual prompts are jointly modeled to improve benign-malignant classification. For our text-only clinical classification task, MP-UDTN was adapted by removing the image encoder and multimodal fusion modules while retaining the text feature extraction and classification components. The model was trained and evaluated using the same data splits and evaluation protocol as the proposed method. Other implementation details, including the optimization strategy and training settings, followed the original implementation when applicable.

**SCL** (33): By introducing supervised contrastive constraints under data-scarce scenarios, this method effectively aligns prompt representations of the same class, thereby significantly enhancing the generalization ability and fine-tuning performance of pre-trained models on downstream few-shot tasks.

**BioMistral** (34): BioMistral is a domain-specific large language model further pre-trained on large-scale biomedical corpora. By effectively leveraging biomedical domain knowledge and learning high-quality semantic representations, it has demonstrated outstanding performance across a variety of biomedical natural language processing tasks. In our experiments, the pretrained BioMistral-7B model was directly used as a zero-shot instruction-based classifier without parameter fine-tuning. The classification task was formulated as a text generation problem through instruction prompting. During inference, beam search with four beams and a temperature of 0.1 were adopted for text generation. The generated responses were normalized and matched to the predefined class labels to obtain the final predictions. The model was evaluated using the same data splits and evaluation protocol as the proposed method to ensure a fair comparison.

### 4.3 Experiment settings

In this experiment, we evaluated the proposed method on two medical short text classification datasets. Multiple training sample-size settings were adopted, and a validation set of the same size was used. To ensure the reproducibility of the experimental results, all experiments were conducted with a fixed random seed.

In the few-shot experimental settings (20-shot, 25-shot, and 30-shot), we employ stratified random sampling to construct labeled training samples, ensuring class balance across all categories. Specifically, *K* samples per class (where *K*∈{20, 25, 30}) are randomly selected from the original training set to form the few-shot training set. Subsequently, an equal number of samples per class are randomly selected from the remaining training data to construct the validation set, while the remaining samples are not used during few-shot training. The test set is kept identical to the official test split and remains fixed across all experiments to ensure fair comparisons under different few-shot settings.

To reduce the influence of randomness, each few-shot setting was repeated using three independent runs with different random seeds, corresponding to different data sampling processes. We report the average performance together with the standard deviation over the three runs in all result tables.

In terms of model and training settings, both the proposed method and baseline approaches were initialized with corresponding pretrained language models for the Chinese and English datasets. Prompt learning was incorporated during training, using a template configuration with template_id = 3 and a kpt1 - type verbalizer. The model was optimized using the AdamW optimizer with a learning rate of 4 × 10^−5^. In addition, supervised contrastive learning was introduced during training, where samples belonging to the same class within a mini-batch were treated as positive pairs, while samples from different classes were regarded as negative pairs.

Four commonly used evaluation metrics were employed to assess model performance, including Accuracy (Acc), Precision (Pre), Recall (Rec), and F1-Score (F1).

The model performance was evaluated using Accuracy (Acc), Precision (Pre), Recall (Rec), and F1-score. Since the datasets exhibit a certain degree of class imbalance, Precision, Recall, and F1-score are reported as macro-averaged metrics unless otherwise specified.

All experiments were conducted on a unified experimental platform equipped with an NVIDIA GeForce RTX 4090 Founders Edition GPU, an Intel(R) Core(TM) i9-10980XE processor operating at 3.00 GHz, and 125 GB of RAM. The experimental environment was implemented using Python 3.9.16 and PyTorch 1.7. Unless otherwise specified, the maximum input sequence length was set to 256 tokens, the batch size was 16, and the maximum number of training epochs was 20. The temperature parameter and the weight of the supervised contrastive loss were set to 0.5 and 1.0, respectively. Early stopping was not adopted during training, and the model obtained after the final training epoch was used for evaluation. To improve the reproducibility of the proposed framework, the hyperparameters were determined through a grid search on the validation set. The final configuration was selected according to the best validation F1-score and was consistently applied to all few-shot settings without further tuning.

### 4.4 Main results

The experimental results of the two datasets are shown in Table 2. For each model, we conducted three repeated runs and reported the average as the final result.

**Table 2:** Results of Acc, Pre, Rec, and F1 (in %) on two datasets.

Datasets	Model	20-shot				25-shot				30-shot			
		Acc	Pre	Rec	F1	Acc	Pre	Rec	F1	Acc	Pre	Rec	F1
zh	TextCNN	53.03±0.11	50.77±0.01	51.43±0.01	49.30±0.08	57.23±0.12	53.29±0.03	57.38±0.04	49.85±0.06	58.91±0.21	51.79±0.05	53.57±0.05	53.57±0.05
	TF-IDF	60.78±0.06	49.87±0.02	49.92±0.05	45.73±0.03	61.34±0.06	50.13±0.02	50.24±0.05	46.14±0.03	62.18±0.06	51.73±0.02	53.81±0.05	48.03±0.03
	ChineseMedical BERT	55.76±0.06	55.88±0.20	50.00±0.03	55.53±0.09	46.55±0.04	44.02±0.04	49.05±0.05	46.40±0.04	56.55±0.12	44.02±0.09	49.05±0.01	46.40±0.04
	LLaMA	68.31±0.23	66.48±0.08	65.02±0.04	64.91±0.10	70.62±0.14	69.56±0.08	73.13±0.05	73.25±0.06	72.00±0.12	68.73±0.01	70.54±0.02	77.31±0.06
	P-Tuning	76.47±0.15	63.47±0.03	77.38±0.03	64.55±0.07	86.55±0.14	72.00±0.02	**86.19**±0.02	75.96±0.05	88.24±0.12	71.67±0.04	71.67±0.04	71.67±0.09
	MP-UDTN	70.38±0.09	72.15±0.04	71.46±0.04	71.58±0.03	73.16±0.15	69.46±0.02	71.57±0.02	70.38±0.07	74.68±0.14	73.53±0.02	72.46±0.02	72.86±0.05
	SCL	77.39±0.09	76.88±0.03	67.89±0.05	67.82±0.05	78.24±0.11	70.76±0.03	72.76±0.02	76.35±0.03	81.51±0.11	73.48±0.05	76.33±0.01	75.18±0.07
	BioMistral	78.24±0.09	74.12±0.04	70.00±0.04	76.88±0.03	75.26±0.15	71.12±0.02	69.00±0.02	74.82±0.07	80.35±0.14	72.12±0.02	75.00±0.02	72.88±0.05
	Ours	**80.75**±0.07	**77.45**±0.21	**82.38**±0.03	**79.60**±0.10	84.95±0.04	**86.52**±0.04	80.95±0.06	**88.55**±0.14	**95.79**±0.12	**89.03**±0.21	**91.42**±0.11	**90.18**±0.05
en	TextCNN	53.02±0.15	47.68±0.03	47.03±0.03	46.21±0.07	58.00±0.14	51.39±0.02	51.66±0.02	50.91±0.05	61.74±0.12	48.27±0.04	48.31±0.04	48.29±0.09
	TF-IDF	46.76±0.06	48.94±0.04	48.60±0.05	43.68±0.05	50.07±0.05	47.52±0.04	46.88±0.04	44.79±0.03	53.02±0.07	49.62±0.07	49.75±0.09	47.33±0.07
	ClinicalBERT	44.97±0.06	49.17±0.04	48.91±0.05	43.37±0.05	51.54±0.03	59.90±0.01	49.94±0.01	50.64±0.03	52.89±0.01	59.90±0.05	59.93±0.07	52.37±0.04
	LLaMA	67.43±0.23	69.14±0.08	68.92±0.00	65.37±0.10	69.43±0.07	61.02±0.21	61.29±0.03	67.38±0.10	67.43±0.04	60.75±0.04	60.94±0.06	66.05±0.04
	P-Tuning	65.77±0.21	68.93±0.05	69.17±0.05	68.67±0.05	71.81±0.03	73.25±0.03	**71.39**±0.04	**79.53**±0.03	71.14±0.12	**75.87**±0.01	73.66±0.01	73.36±0.05
	MP-UDTN	62.27±0.12	65.64±0.04	64.58±0.02	67.27±0.03	68.47±0.11	62.58±0.00	68.85±0.01	66.68±0.08	70.47±0.12	72.58±0.03	71.85±0.04	72.56±0.06
	SCL	60.20±0.11	59.67±0.00	59.32±0.01	61.57±0.08	67.11±0.12	69.50±0.03	64.25±0.04	66.75±0.06	73.17±0.11	72.91±0.03	69.95±0.04	68.05±0.06
	BioMistral	**73.83**±0.14	52.59±0.12	50.01±0.22	44.85±0.15	72.93±0.19	45.69±0.04	48.81±0.15	43.00±0.05	73.38±0.11	51.35±0.03	49.41±0.22	43.97±0.13
	Ours	70.47±0.09	**71.20**±0.04	**71.52**±0.05	**70.27**±0.05	**74.49**±0.12	**77.84**±0.13	71.36±0.02	77.34±0.03	**75.17**±0.11	70.58±0.31	**75.40**±0.21	**72.91**±0.22

Bold values indicate the best result.

The experimental results show that our proposed method achieves the best performance on both datasets and significantly outperforms other baseline models across multiple metrics, demonstrating its effectiveness and stability for few-shot medical text classification. Overall, as the number of training samples increases, the performance of all compared methods improves to varying degrees, indicating that additional labeled data can effectively alleviate the generalization limitations in few-shot learning. However, compared with other approaches, our method exhibits a more pronounced performance gain as the sample size grows. In particular, it achieves higher overall performance under the 30-shot setting, suggesting that incorporating contrastive learning can further enhance feature representations and improve the robustness and generalization ability of the model in few-shot scenarios.

Further analysis of different types of models reveals that traditional deep learning classifiers perform relatively poorly under few-shot settings, mainly because they lack the support of pre-trained knowledge and therefore struggle to learn stable and effective semantic representations from limited data. In contrast, methods based on pre-trained language models generally achieve stronger baseline performance, although their effectiveness is highly influenced by task matching and data scale. The domain-specific medical pre-trained model performs worse than our method, which may be due to its tendency to overfit in few-shot scenarios or a mismatch between its pre-training objectives and the discriminative requirements of our task, thus limiting its transferability.

To further ensure the comprehensiveness of the comparison, we additionally introduce four representative baseline methods, including TF-IDF, MP-UDTN, SCL, and BioMistral. TF-IDF relies on sparse lexical features and lacks contextual semantic modeling capability, leading to limited performance in few-shot settings. MP-UDTN improves representation learning through domain-specific neural architectures; however, its dependence on task-specific training still limits its generalization ability. SCL introduces supervised contrastive learning to enhance feature discriminability, but its effectiveness is still constrained under extremely limited training data. BioMistral, as a biomedical pre-trained language model, possesses strong prior knowledge, but may suffer from task mismatch and insufficient adaptation in few-shot classification scenarios.

In addition, prompt learning methods can better activate the latent knowledge of pre-trained models compared to direct fine-tuning, leading to more stable improvements under few-shot settings. However, the experimental results indicate that prompt learning alone is still insufficient to fully capture discriminative information among samples. By contrast, our method further integrates contrastive learning on top of prompt learning. Through sample-level semantic alignment and discriminative constraints, it can effectively pull representations of the same class closer while pushing representations of different classes farther apart, thereby significantly improving the model's discriminative capability and generalization performance under data-scarce conditions. In summary, the main experimental results demonstrate that the combination of prompt learning and contrastive learning enables superior performance on both datasets and provides a more effective solution for few-shot medical text classification.

To provide a more comprehensive evaluation of the proposed method under class-imbalanced settings, we further present the confusion matrices together with the per-class Precision, Recall, and F1-score on the test sets of both datasets, as shown in Table 3. These class-wise results provide a more detailed assessment of the model performance on both the majority and minority classes, complementing the overall evaluation metrics and further demonstrating the reliability of the proposed method for clinical decision-support.

**Table 3:** Confusion matrices and per-class classification performance of the proposed method on the test sets of the two datasets.

Dataset	True Label	Predicted Non-SDB	Predicted SDB	Precision (%)	Recall (%)	F1-score (%)
Medical_zh	Non-SDB	12	2	80.00	85.71	82.76
	SDB	3	102	98.08	97.14	97.61
Medical_en	Non-SDB	30	9	51.72	76.92	61.86
	SDB	28	82	90.11	74.55	81.59

For the Medical_en dataset, the Non-SDB category achieved a relatively lower precision compared with the SDB category. This indicates that some SDB samples were incorrectly classified as Non-SDB, which may be associated with the imbalanced class distribution and the limited representation of Non-SDB samples in the dataset. Nevertheless, the model maintained a satisfactory recognition capability for the Non-SDB category, indicating its ability to capture discriminative patterns across different clinical categories. Overall, the proposed framework achieved relatively balanced performance across different classes, demonstrating its potential for supporting SDB diagnosis under few-shot settings.

### 4.5 Ablation study

To evaluate the individual contributions of prompt learning, the Knowledgeable Verbalizer (KV), and supervised contrastive learning in the proposed framework, we conducted a series of ablation experiments, and the corresponding results are presented in Table 4. Specifically, *w/o Contrastive* denotes the variant that removes the supervised contrastive learning objective while retaining the prompt-based learning framework. In contrast, *w/o Prompt* represents the variant in which the original medical text is directly fed into the pretrained language model without prompt reformulation, while the supervised contrastive learning module is preserved for representation optimization. Furthermore, *w/o KV* denotes the variant in which the Knowledgeable Verbalizer is replaced with a standard verbalizer without knowledge enhancement, while all other components remain unchanged.

**Table 4:** Ablation study results on zh and en datasets.

Dataset	Method	Acc	F1
zh	Ours	0.9579	0.9018
	w/o Contrastive	0.6218	0.5040
	w/o Prompt	0.6386	0.5358
	w/o KV	0.5218	0.5503
en	Ours	0.7517	0.7291
	w/o Contrastive	0.6577	0.5914
	w/o Prompt	0.5503	0.5029
	w/o KV	0.5029	0.5297

The complete framework consistently achieves the best performance on both datasets, demonstrating the effectiveness of jointly integrating prompt learning, the Knowledgeable Verbalizer (KV), and supervised contrastive learning.

The results indicate that prompt learning can effectively improve task adaptation capability under few-shot settings by leveraging semantic priors embedded in pretrained language models. Furthermore, the Knowledgeable Verbalizer enhances semantic alignment between class labels and clinically relevant medical concepts by introducing domain-specific knowledge into the prediction space, thereby improving the interpretability and discriminative capability of prompt-based classification. Meanwhile, supervised contrastive learning further enhances the discriminability of semantic representations by improving intra-class compactness and inter-class separability in the embedding space. The ablation results show that removing any of these components leads to a noticeable performance degradation, confirming that each module contributes positively to the overall framework. Therefore, the collaborative optimization of these three components contributes to more robust and generalized performance for few-shot medical text classification.

### 4.6 Analysis of prompt engineering strategies

To further improve the interpretability and clinical relevance of the proposed framework, we analyze the influence of prompt templates and knowledgeable verbalizer configurations on the experimental results. Specifically, prompt templates guide the pre-trained language model to focus on clinically relevant semantic information by reformulating the classification task as a masked language modeling problem, while the knowledgeable verbalizer incorporates domain-specific medical knowledge by establishing semantically meaningful mappings between class labels and representative medical concepts. The following analyses demonstrate how these two components contribute to the prediction process and provide further insight into the effectiveness of the proposed framework.

#### 4.6.1 Analysis of prompt templates

To better align the downstream SDB diagnosis task with the masked language modeling objective of the PLMs, four handcrafted prompt templates were manually designed based on common clinical expressions, symptom descriptions, and disease assessment patterns frequently appearing in medical records, which are presented in Table 5. By reformulating the classification task into a masked token prediction problem, these templates explicitly guide the model to infer the patient's disease status while incorporating domain-specific medical knowledge into the prompt construction.

**Table 5:** Handcrafted prompt templates evaluated in this study.

ID	Chinese template	English template
1	该患者的诊断结果为[MASK]°	The patient's diagnosis is [MASK].
2	该患者的病情为[MASK]°	The patient's condition is classified as [MASK].
3	该患者的临床表现为[MASK]°	The patient's condition is [MASK].
4	综合评估,该患者属于[MASK]°	Based on the assessment, the patient belongs to [MASK].

**Figure 2:**
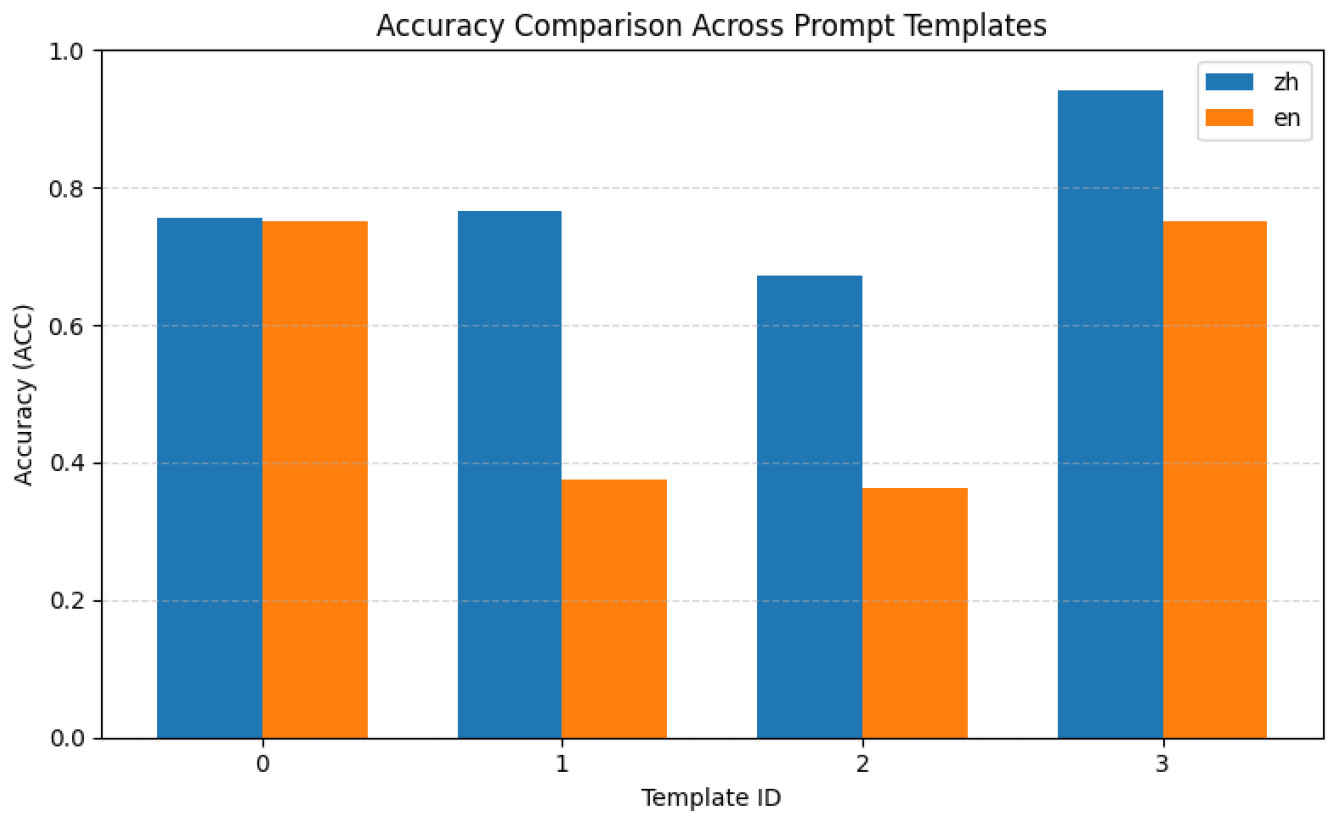
Accuracy comparison across different prompt templates.

The experimental results are shown in Figure 2. The results demonstrate that the construction strategy of prompt templates can significantly influence the semantic representation capability of pretrained language models as well as downstream classification performance under few-shot settings.

On both the Chinese and English datasets, Template ID=3 achieved the best classification performance, indicating that appropriately designed prompt structures can more effectively establish semantic mappings between medical texts and target labels, thereby enhancing the model's capability to capture clinically relevant semantic information.

In addition, compared with the English dataset, the Chinese dataset exhibited more pronounced performance fluctuations under different template settings. A possible reason is that Chinese medical texts generally contain stronger contextual dependencies and more complex implicit semantic expressions, making the model more sensitive to prompt structures under few-shot conditions. Therefore, appropriate prompt template design plays an important role in improving the stability and generalization capability of medical text classification models.

Overall, the experimental results further verify the critical role of prompt templates in guiding task adaptation and semantic representation learning for pretrained language models, and demonstrate that well-designed prompt structures can effectively improve the performance of few-shot medical text classification tasks. These observations indicate that the proposed framework is sensitive to prompt formulation, highlighting the importance of prompt engineering in few-shot medical text classification. The performance differences across prompt templates further justify the selection of Template ID=3 as the default configuration in the proposed framework.

#### 4.6.2 Analysis of knowledgeable verbalizer configurations

Instead of manually assigning label words to each category, the proposed framework adopts a knowledgeable verbalizer to establish the mapping between class labels and semantically related medical concepts. Domain-specific medical knowledge is incorporated by selecting representative medical terms that are highly relevant to each category, enabling the model to better align the prediction space with clinically meaningful semantics. Representative label words associated with different categories in the knowledgeable verbalizer are shown in Table 6.

**Table 6:** Representative label words associated with different diagnostic categories in the knowledgeable verbalizer.

Dataset	Class label	Representative label words
Medical_zh	SDB-Positive	重度, 呼吸暂停, 低氧, 异常, 严重
	SDB-Negative	正常, 轻度, 健康, 无异常, 良好
Medical_en	SDB-Positive	severe, apnea, abnormal, hypoxia, positive
	SDB-Negative	normal, mild, healthy, negative, unremarkable

To further evaluate the influence of different KPT (Knowledgeable Prompt Tuning) configurations on medical text classification performance, we conducted comparative experiments on multiple KPT variants under the 30-shot setting. The results are reported in Table 7.

**Table 7:** Performance comparison of different KPT configurations on two datasets.

Dataset	KPT Config	Acc	F1
zh	kpt	0.6723	0.5134
	kpt1	0.9579	**0.9018**
	kpt2	0.8824	0.7896
	kpt3	0.8487	0.7398
	kpt4	0.9412	0.8436
	kpt5	0.9328	0.8556
en	kpt	0.6483	0.6989
	kpt1	**0.7517**	0.7291
	kpt2	0.6161	0.6152
	kpt3	0.6779	0.6862
	kpt4	0.7111	**0.7314**
	kpt5	0.6846	0.6902

The best results are highlighted in bold.

Overall, different KPT configurations have a significant impact on model performance. Among them, *kpt1* demonstrates more stable and competitive performance on both the Chinese and English datasets, indicating that an appropriate knowledge-enhanced strategy can effectively improve semantic modeling and classification capability in few-shot scenarios.

On the Chinese dataset, although some configurations achieve comparable accuracy, their F1-scores still differ, suggesting that different KPT strategies exhibit different preferences in balancing precision and recall. On the English dataset, the performance discrepancies among different configurations are more pronounced, which indicates that English medical text classification is more sensitive to the knowledge injection strategy and label semantic formulation. This further highlights the importance of knowledge enhancement in cross-lingual settings.

Further analysis of the label words selected by KPT shows that better-performing configurations tend to choose more domain-specific medical terms, such as clinical severity grading expressions, rather than more generic words. This domain-aligned label semantic mapping can strengthen the model's understanding of medical contexts, thereby improving classification robustness and reliability.

These findings demonstrate that *kpt1* is a strong and robust configuration across both datasets, and further confirm that knowledge-enhanced strategies tailored to medical text characteristics can provide more stable and effective performance gains under few-shot learning settings.

### 4.7 Computational cost analysis

To evaluate the computational efficiency of the proposed framework, we analyze its computational cost, GPU memory consumption, training time, and inference efficiency. As shown in Table 8, all experiments are conducted on an NVIDIA RTX 4090 GPU with 24 GB memory. The results indicate that the proposed method requires only moderate GPU memory while maintaining efficient performance during both training and inference.

**Table 8:** Computational cost of the proposed framework on the *zh* and *en* datasets.

Metric	zh dataset	en dataset
Peak GPU Memory (GB)	9.46	9.98
Training Time / Epoch (s)	0.73	0.58
Inference Time / Sample (ms)	1.90	2.28

This efficiency mainly stems from the lightweight design of prompt learning and the knowledgeable verbalizer, which do not introduce additional complex network structures or significant computational burden. Overall, the proposed framework achieves a good balance between performance and efficiency, demonstrating its feasibility for deployment in real-world clinical scenarios.

### 4.8 Case study

To improve interpretability and clinical relevance, we present a case study in Table 9 to analyze how the proposed method makes predictions.

**Table 9:** Case study of interpretability analysis.

Evidence	Clinical meaning	Influence on prediction
Obesity (BMI = 32.1)	Major risk factor for OSA	Strong positive signal
Loud snoring + apnea episodes	Core diagnostic symptoms	Primary decision trigger
Hypertension	Common comorbidity of OSA	Supports severity assessment
Oxygen desaturation	Severe nocturnal hypoxemia	Strong severity indicator

For this patient, the model correctly predicts severe obstructive sleep apnea-hypopnea syndrome. The decision is primarily driven by several clinically meaningful indicators, including obesity, typical sleep apnea symptoms such as loud snoring and apnea episodes, hypertension, and severe nocturnal oxygen desaturation. From an interpretability perspective, these features correspond to well-established clinical diagnostic criteria for sleep-disordered breathing. In particular, obesity and nocturnal hypoxemia are strong risk factors, while snoring and apnea episodes are direct symptomatic evidence. Furthermore, the prompt design guides the model to focus on respiratory and cardiovascular-related symptoms, while the verbalizer strengthens the mapping between symptom descriptions and diagnostic categories. This ensures that the prediction is not purely statistical, but aligned with medical reasoning.

Overall, this case demonstrates that the proposed method provides clinically interpretable decision evidence, improving transparency and potential trustworthiness in real-world medical applications.

### 4.9 Discussion and limitations

The proposed framework achieves promising performance under the few-shot setting, which can be attributed to several factors. First, prompt learning reformulates the downstream classification task into a masked language modeling objective, enabling the pretrained language model to effectively leverage its prior medical knowledge. Second, the knowledgeable verbalizer establishes semantically meaningful associations between class labels and domain-specific medical concepts, thereby improving semantic alignment during prediction. Finally, by effectively integrating heterogeneous clinical information through prompt-based learning, the proposed framework is able to capture clinically meaningful semantic patterns even with limited annotated samples. These factors jointly contribute to the robust performance of the proposed framework.

Although the proposed framework achieves promising performance on both the self-constructed zh dataset and the publicly available en dataset, this study still has some limitations. First, the current evaluation is conducted on only two datasets. Further validation on external datasets collected from different clinical institutions or domains would further demonstrate the generalization capability and practical applicability of the proposed framework. Second, variations in clinical documentation styles, annotation protocols, and patient populations across institutions may influence model performance. Therefore, future work will focus on validating the proposed framework on more diverse cross-institutional and cross-domain clinical datasets, as well as investigating domain adaptation techniques to further improve its robustness and generalization ability in real-world clinical environments.

In addition, due to the characteristics of the self-constructed zh dataset, there may exist certain dataset-specific biases during model training, which could lead to dependence on particular data distributions. Meanwhile, the proposed framework relies on manually designed prompts and knowledgeable verbalizers; therefore, it may be sensitive to prompt formulations and label semantics, potentially resulting in performance fluctuations under different configurations. Similar to most few-shot learning methods, the model may occasionally produce incorrect predictions when handling ambiguous or highly similar clinical descriptions, mainly due to decision uncertainty under limited contextual information.

In future work, we will explore automated prompt optimization, uncertainty modeling, and more robust knowledge integration strategies to further improve the stability and clinical reliability of the proposed framework.

## 5 Conclusions

In this paper, we proposed a few-shot diagnosis framework for Sleep-Disordered Breathing (SDB) based on medical text, named SDB-FL. By reformulating the downstream classification task into a prompt-based masked language modeling task, the proposed method leverages prompt templates and knowledgeable verbalizers to effectively activate latent medical semantic knowledge embedded in pre-trained language models. In addition, supervised contrastive learning is introduced to enhance the discriminative ability of semantic representations and improve the generalization capability of the model. Experimental results on two medical datasets demonstrate that the proposed method consistently outperforms several baseline methods under different few-shot settings, verifying its effectiveness and robustness for few-shot medical text classification tasks.

Although the proposed method achieves promising performance, several limitations still remain. For example, the current datasets are relatively limited in scale, the study only focuses on binary classification tasks, and the prompt templates still rely on manual design. In future work, multimodal clinical information and automatic prompt construction strategies will be further explored to improve the diagnostic capability and generalization performance of the model in real-world clinical scenarios.

## Data Availability

The original contributions presented in the study are included in the article/Supplementary material, further inquiries can be directed to the corresponding authors.

## Footnotes

1
**https://www.kaggle.com/datasets/ziya07/sleep-disordered-breathing-detection**
